# Genome Analysis of *Legionella pneumophila* Strains Using a Mixed-Genome Microarray

**DOI:** 10.1371/journal.pone.0047437

**Published:** 2012-10-18

**Authors:** Sjoerd M. Euser, Nico J. Nagelkerke, Frank Schuren, Ruud Jansen, Jeroen W. Den Boer

**Affiliations:** 1 Regional Public Health Laboratory Kennemerland, Haarlem, The Netherlands; 2 Department of Community Medicine, United Arab Emirates University, Al-Ain, United Arab Emirates; 3 TNO Microbiology and Systems Biology, Zeist, The Netherlands; University of Louisville, United States of America

## Abstract

**Background:**

*Legionella*, the causative agent for Legionnaires’ disease, is ubiquitous in both natural and man-made aquatic environments. The distribution of *Legionella* genotypes within clinical strains is significantly different from that found in environmental strains. Developing novel genotypic methods that offer the ability to distinguish clinical from environmental strains could help to focus on more relevant (virulent) *Legionella* species in control efforts. Mixed-genome microarray data can be used to perform a comparative-genome analysis of strain collections, and advanced statistical approaches, such as the Random Forest algorithm are available to process these data.

**Methods:**

Microarray analysis was performed on a collection of 222 *Legionella pneumophila* strains, which included patient-derived strains from notified cases in the Netherlands in the period 2002–2006 and the environmental strains that were collected during the source investigation for those patients within the Dutch National *Legionella* Outbreak Detection Programme. The Random Forest algorithm combined with a logistic regression model was used to select predictive markers and to construct a predictive model that could discriminate between strains from different origin: clinical or environmental.

**Results:**

Four genetic markers were selected that correctly predicted 96% of the clinical strains and 66% of the environmental strains collected within the Dutch National *Legionella* Outbreak Detection Programme.

**Conclusions:**

The Random Forest algorithm is well suited for the development of prediction models that use mixed-genome microarray data to discriminate between *Legionella* strains from different origin. The identification of these predictive genetic markers could offer the possibility to identify virulence factors within the *Legionella* genome, which in the future may be implemented in the daily practice of controlling *Legionella* in the public health environment.

## Introduction

The bacterium *Legionella* is the causative agent for Legionnaires’ disease, an acute pneumonia that accounts for a significant amount of community-acquired pneumonias (ranging from 1.9–20%) [1]–[3], and proves fatal in about 6–8.5% of diagnosed cases [4], [5]. *Legionella* is ubiquitous in both natural and man-made aquatic environments, and the major route of transmission is inhalation of the bacterium that is spread into the air as an aerosol from its reservoir [6]. A wide range of contaminated water systems have been identified as the source of infection for Legionnaires’ disease patients in numerous outbreak investigations, including cooling towers, saunas, and whirlpool spas. Genetic comparisons of the clinical and the environmental *Legionella* strains form an essential part of these investigations [7], [8], although interpretations are often made without full understanding of the underlying distribution of genotypes in clinical and environmental strain populations [9].

In the Netherlands, a National *Legionella* Outbreak Detection Programme (NLODP) [10] was installed in 2002, which aimed to shorten response time between diagnosis of patients and source identification, and to improve source investigation and elimination. Together with the implementation of new governmental laws and guidelines to prevent growth of *Legionella* bacteria in potential sources, it was attempted to diminish the overall impact of Legionnaires’ disease in the Netherlands. Nevertheless, despite these excessive efforts the incidence of Legionnaires’ disease has only increased since 1999 [11].This unexpected trend might partly be due to the unfocussed broad scope of preventive measures that do not take virulence factors into account [12]–[14]. Previous studies have shown that the majority (>90%) of Legionnaires’ disease cases are caused by the species *Legionella pneumophila*, and about 80% more specifically by *L. pneumophila* serogroup 1 [15], [16]. However, the distribution of genotypes within these clinical strains is significantly different from the distribution found in environmental strains [16]–[18]. These findings suggest a discrepancy in virulence between genotypes with a possible genetic base for these differences [12], which is in line with results from the multigenome analysis of 249 *Legionella* strains that was performed by Cazalet et al. [19]. The development of novel genotypic methods that offer the ability to distinguish clinical from environmental strains could form a welcome next step in focusing more on relevant (virulent) *Legionella* species in control efforts.

In a previous study, we described the development of a mixed-strain microarray using comparative genome hybridization (CGH), that contained genetic data from both clinical and environmental strains [12]. A supervised statistical analysis using Genetic Programming was used to identity DNA markers that could discriminate between clinical and environmental *Legionella* strains, and a model consisting of five markers was developed to predict the origin of a strain: clinical or environmental. The final model correctly predicted 100% of the clinical strains and 69% of the environmental strains [12]. Despite these promising results, there might be other methodological approaches that could lead to (at least) comparable predictive performances. Potentially, geographical differences in virulence may have influenced the previous analysis [5], as clinical strains from Dutch patients who stayed abroad during their incubation period were included in the strain collection. In this study we have explored these possibilities.

We would like to improve these results by using more strict inclusion criteria for the strain collection, using continuous microarray data instead of binary data, and exploring alternative statistical approaches. Therefore, we here present a novel approach using the microarray data of the *Legionella* strains that were generated by Yzerman et al., to develop a new prediction model that can appropriately discriminate between clinical and environmental strains using a minimal number of DNA markers. This prediction model was based on the Random Forest algorithm [20], [21], which is well suited for the use of microarray data in the prediction of the origin of strains using a small set of DNA markers [20].

## Methods

### Strain Collection

The strain collection that was described by Yzerman et al. [12], was also used for the present analyses. This collection encompasses patient-derived strains from notified cases in the Netherlands in the period from 2002–2006 and the environmental strains that were collected during the source investigation for those patients. Together, microarray data were available for 257 unique *Legionella* strains.

For our analyses, we excluded the clinical strains (n = 34) that were derived from patients who had stayed abroad for ≥5 days during their incubation period of 2–10 days (as they might have been infected by a source situated abroad), resulting in a more correct comparison with Dutch environmental strains. Additionally, we excluded the clinical strain (n = 1) from a patient who had stayed in a hospital during the incubation period, as nosocomial Legionnaires’ disease is likely to occur due to less virulent strains [22]. This resulted in a strain collection of 222 unique *Legionella* strains (49 clinical strains and 173 environmental strains) that were used in the present study (File S1).

### Microarray Development

The development of the mixed-genome microarray has been described elsewhere [12]. In short, eight *L. pneumophila* strains were selected based on their diversity (both clinical and environmental strains were used) to provide a shotgun library. The microarray consisted of 3360 genomic fragments and was used to analyze the genomic composition of the 222 *Legionella* strains in our collection, by comparing labeled DNA from each strain with the library. The data for all spots were calculated as ratios between the tester strain and the reference strains.

The number of markers was reduced based on the observation that approximately 80% of the 3360 markers were present in all strains and therefore apparently encompassed the *L. pneumophila* core genome. The remaining 20% did show variation in presence between individual strains. Additionally, the number of relevant markers was further reduced by only selecting a limited number of representatives in those cases where multiple markers showed nearly identical patterns over the complete data set (suggesting partial overlap or close linkage in the genome). This resulted in a selection of 480 potentially relevant markers that were used in the further development of the prediction model [12].

For all of these 480 markers, Yzerman et al., determined an individual marker-dependent cut-off value to convert the linearly distributed ratios into binary values that represent the presence or absence of each marker [12]. As Random Forest allows the use of continuous predictor variables, we chose to use the continuous microarray data (ratios) to make a selection of DNA markers to enter in our prediction model.

### Methodological Approaches for Marker Selection

The selection of a minimum number of relevant genetic markers that together achieve good predictive performance is one of the challenges in most gene expression studies [20]. Several methodological approaches that can cope with the high-dimensionality (more variables than observations) and noisiness of the data generated in microarray studies have been developed in recent years [23]. However, many of these approaches are not appropriate when the main objective is to obtain the smallest set of genetic markers [20], [23].

Random Forest is an algorithm for classification that uses an ensemble of different classification trees [20], [21]. Every single classification tree is built using a bootstrap sample of the data, and at each split in the tree the candidate set of variables is a random subset of the variables. This means that Random Forest uses both bagging (bootstrap aggregation) and random variable selection for tree building, which results in low correlation of the individual trees [20]. The Random Forest method has excellent performance in classification tasks, and several characteristics that make it well suited for the analyses of microarray data, including the following: (1) it can handle many more variables than observations; (2) it has good predictive performance even when most predictive variables are noise; (3) it does not overfit; and (4) it can handle a mixture of categorical and continuous predictors [20]. Taking this all in consideration, we chose the Random Forest method to select a minimal set of predictive markers for the *L. pneumophila* strain collection.

### Marker Selection for *L. pneumophila* Strains

Although Random Forest itself makes use of cross-validation, we decided to increase the level of cross-validation as follows. The *L. pneumophila* strain collection in total consisted of 49 clinical isolates and 173 environmental strains. These strains were randomly assigned to 10 different training datasets, each consisting of 24 clinical strains and 87 environmental strains. For all 10 training datasets Random Forest was used to select the 25 markers with the highest rank of “importance” in the prediction of the origin of the strains (clinical or environmental). From these 250 selected markers (25 markers per training dataset) we selected the 25 genetic markers that were present in the majority of the 10 training datasets (File S2).

An eleventh training dataset was randomly constructed consisting of 24 clinical and 87 environmental strains. A logistic regression model (PASW release 18.0, IBM SPSS inc., New York, NY) was developed (forward logistic regression) with the 25 genetic markers entered as independent variables, and the origin of the strains (clinical or environmental) as dependent variable (Files S3 and S4). The choice of using logistic regression was based on the ease of adjusting this model (by changing the intercept parameter only) when prior probabilities in datasets vary. Although the followed methodological approach in general aimed to develop a correct prediction of the origin of strains with a minimal set of DNA markers, the main goal of the prediction model was the correct prediction of the clinical isolates. The incorrect prediction of a clinical isolate (false-negative) could potentially form a future risk in the public health environment, when this model will be further implemented in intervention strategies. The predictive cut-off value for the predicted probabilities was therefore adjusted to maximize the number of correctly predicted clinical isolates, while keeping the number of incorrectly predicted environmental isolates as low as possible. The predictive performance of this model was then tested with a dataset consisting of those strains that were not present in the eleventh training dataset (25 clinical strains and 86 environmental strains), the so-called test dataset (File S5).

The performance of the final prediction model is presented in 2×2 tables, with the corresponding calculations of sensitivity, specificity, negative predictive value (NPV), and positive predictive value (PPV). In public health interventions, and especially in laboratory screening methods, a high NPV is an important performance characteristic [24], [25], and this was another reason why we attempted to correctly predict as many of the clinical isolates as possible.

### Functionality of Markers

The sequences of the four markers that were selected in the final prediction model were compared with all sequences present in the NCBI database (http://www.ncbi.nlm.nih.gov) using BlastN and BlastX. These sequences included the whole genomes of *Legionella longbeachae* (strain NSW150) [26] and *L. pneumophila* strains Paris [27], Philadelphia [28], Lens [27], Corby [29], Alcoy [30], 130b [31], Lorraine [32], and HL 0604 1035 [32], and ATCC 43290 [33].

## Results

**Figure 1:**
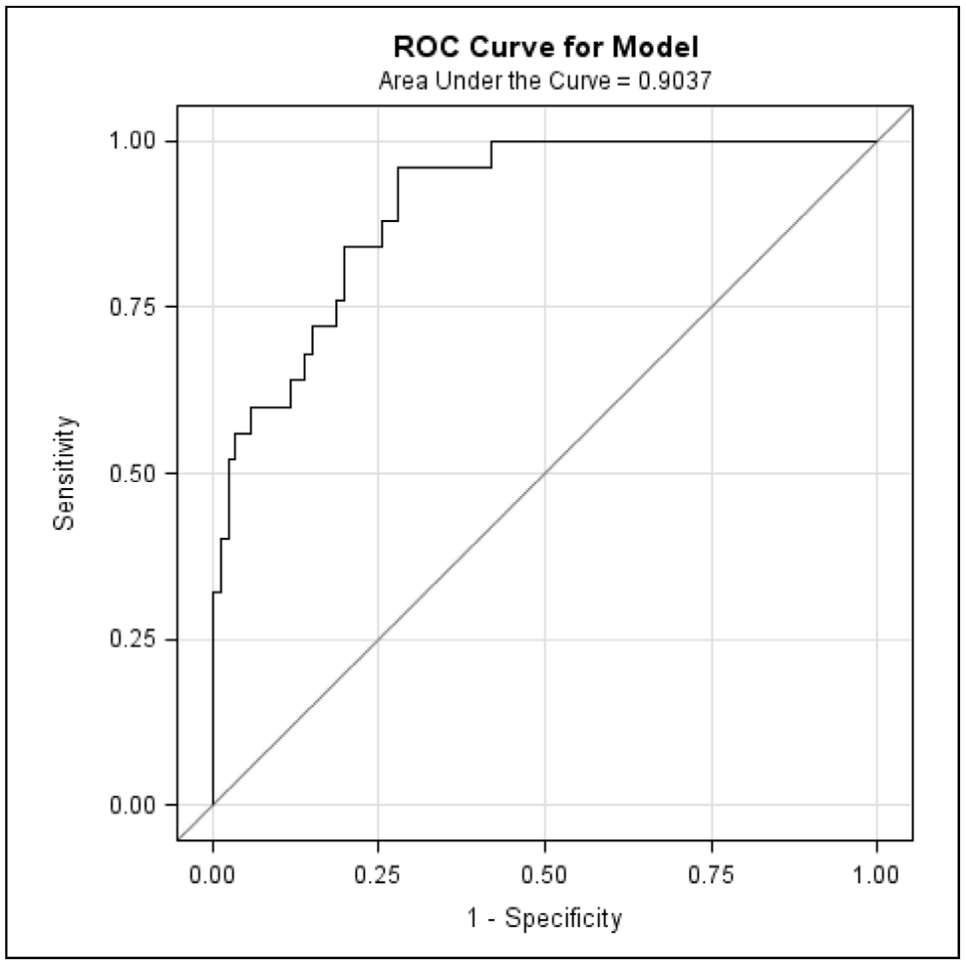
Receiver operating characteristic (ROC) curve for the eleventh test dataset (n = 111). This figure shows the performance of the logistic regression model with the four predictive markers (7B8, 15D6, 16E4, 33F8) for the eleventh test dataset. The proportion of the predicted clinical strains out of the truly clinical strains (true positive rate or sensitivity) is plotted against the proportion of the predicted clinical strains out of the truly environmental strains (false positive rate or 1-specificity).

Within the 250 markers with the highest rank of “importance” that were selected by the Random Forest analyses of the 10 randomly selected training datasets, 51 markers were present more than once. The 25 genetic markers that were present in the majority of the 10 training datasets were entered in the logistic regression model. These logistic regression analyses resulted in the selection of four DNA markers for the final model. In order to attempt to maximize the sensitivity of the model (predict as many of the clinical strains correctly), while minimizing the loss of specificity, we used an arbitrary cut-off value of 0.06 to translate the logistic regression prediction of the training dataset into a classification of the clinical and environmental strains. The 111 strains in the eleventh training dataset were predicted with a sensitivity of 96%, a specificity of 75%, a PPV of 51%, and a NPV of 98% (Table 1). The 111 strains in the eleventh test dataset that were not used for building the logistic regression model were additionally analyzed, and were predicted with a sensitivity of 96%, a specificity of 66%, a PPV of 45%, and a NPV of 98% (Table 2). Figure 1 shows the receiver operating curve of the logistic regression model predictions for the eleventh test dataset. The area under the curve (AUC) or c-statistic was 0.9037.

**Table 1:** Prediction results for the 111 strains in the eleventh training dataset.

Training dataset	Origin of strains		Total
	Clinical	Environmental	
Prediction clinical	23	22	45
Prediction environmental	1	65	66
Total	24	87	111
Sensitivity	96%		
Specificity	75%		
PPV	51%		
NPV	98%		

PPV = positive predictive value; NPV = negative predictive value.

**Table 2:** Prediction results for the 111 strains in the eleventh test dataset.

Test dataset	Origin of strains		Total
	Clinical	Environmental	
Prediction clinical	24	29	53
Prediction environmental	1	57	58
Total	25	86	111
Sensitivity	96%		
Specificity	66%		
PPV	45%		
NPV	98%		

PPV = positive predictive value; NPV = negative predictive value.

The sequence analyses of the four selected markers showed that all four markers were present in one or more of the completely sequenced *Legionella* isolates (Table 3). One of these markers (16E4) is probably related to cell wall synthesis (N-acetylneuraminate synthase), another (33F8) to cellular transport (Xylose symporter). Furthermore, marker 16E4 lies within the lipopolysaccharide synthesis (LPS) region that was identified by Cazalet et al. (2008) as specific for *L. pneumophila* serogroup 1 strains [19]. We additionally checked the eight *L. pneumophila* strains that were used to develop the microarray and could confirm that all four selected markers were present in one or more of these strains. This diminishes the possibility that the selection of the four markers was influences by technical errors or contamination.

**Table 3:** Sequence location of the four predictive markers in ten available *Legionella* genomes.

Legionella strains	Predictive markers			
	7B8	15D6	16E4	33F8
Paris	n/a	n/a	915834–916597	1812491–1813032
Philadelphia	n/a	n/a	823175–823938	1828523–1827989
Lens	n/a	n/a	895789–896552	1794085–1793544
Corby	n/a	n/a	931999–932762	1898600–1898059
Alcoy	n/a	n/a	919333–920096	1893679–1893138
130b	n/a	n/a	899720–900483	1786224–1785683
Lorraine	713281–713837	2631852–2632252	856273–857036	1732233–1732774
HL 060401035	n/a	n/a	882699–883462	1928802–1929343
ATCC 43290	n/a	n/a	825518–826281	1744381–1744915
L. longbeachae NSW150	n/a	n/a	n/a	2612495–2611967

n/a = not applicable.

## Discussion

We have successfully developed a model to predict the origin of *L. pneumophila* strains (clinical or environmental) using mixed-genome microarray data. In this model we used the Random Forest algorithm combined with a logistic regression model to select four genetic markers that correctly predicted 96% of the clinical strains and 66% of the environmental strains that were collected within the Dutch National *Legionella* Outbreak Detection Programme. The negative predictive value (NPV) of the model was relatively high (98%) which corresponds to the criteria for an adequate screening test in public health intervention that were suggested in previous studies on other pathogens (MRSA, ESBL) [24], [25].

Compared with our previous study where we used Genetic Programming to select genetic markers to classify *L. pneumophila* strains in clinical and environmental strains, the present Random Forest model shows a similar performance with respect to sensitivity (100% for Genetic Programming vs. 96% for Random Forest), specificity (69% vs. 66%), PPV (49% vs. 45%), and NPV (100% vs. 98%). However, the five genetic markers that were selected by Genetic Programming differed from the four markers that were selected by Random Forest, which complicates the interpretation of these findings.

One of the differences between the Random Forest method and the Genetic Programming was the use of the continuous microarray data, without binarization (dichotimization) based on a somewhat arbitrary marker-dependent cut-off value. The Random Forest algorithm is well suited for the use of many continuous predictor variables [20], and allowed us to use all available discriminatory power that was present in the data for our prediction model. Furthermore, the more stringent selection criteria for the clinical isolates in our strain collection may have resulted in another set of predictive markers in the model compared to the previous study [12], although a detailed analysis of the reasons why different approaches lead to different marker selection still has to be made. The exclusion of isolates that were derived from patients who have possibly been infected outside the Netherlands, may have diminished the influence of regional differences in genotypic variation between the Dutch environmental isolates, and isolates from abroad. The prediction of the Random Forest prediction model is therefore based on the genotypic variation between clinical and environmental isolates that are representing the situation within the Netherlands, and not in other countries. Further studies using strain collection from other geographic regions, and collected in different time periods could help to determine the influence of these factors on the genotypic variation.

The analysis of the sequences of the four identified markers suggests that there are several possible biological pathways that could underlie their predictive value. The identification of marker 16E4 that lies within the previously reported LPS cluster [19] may indicate the importance of this region for further investigation of a genetic base for virulence differences between *Legionella* strains.

The identification of predictive genetic markers with the Random Forest model offers the possibility to further study virulence factors within the *Legionella* genome, which in the future may even be implemented in the daily practice of controlling *Legionella* in the public health environment. For instance, appropriate control procedures could either be timely implemented or abandoned, based on the potential risk of the *Legionella* strains found in water samples during regular sampling investigations, although other factors such as the population at risk should always be taken into account as well.

## Supporting Information

File S1 **List of strains used in this study.**
(TXT)

File S2 **List the genetic markers selected by the Random Forest algorithm.**
(TXT)

File S3 **The logistic regression model.**
(TXT)

File S4 **Microarray data from the 111 strains in the 11^th^ training dataset.**
(TXT)

File S5 **Microarray data from the 111 strains in the 11^th^ test dataset.**
(TXT)
