# Correction: Genome Analysis of *Legionella pneumophila* Strains Using a Mixed-Genome Microarray

**DOI:** 10.1371/annotation/18f57a8b-22a4-4251-ad3a-f1b1b8041011

**Published:** 2013-06-13

**Authors:** Sjoerd M. Euser, Nico J. Nagelkerke, Frank Schuren, Ruud Jansen, Jeroen W. Den Boer

In Table 3, the sequence locations of marker 7B8 and marker 15D6 in the Lorraine strain have been switched.

The correct sequence location for marker 7B8 in the Lorraine strain is: 2631852–2632252.

The correct sequence location for marker 15D6 in the Lorraine strain is: 713281–713837. 

